# Spastin Promotes the Migration and Invasion Capability of T98G Glioblastoma Cells by Interacting with Pin1 through Its Microtubule-Binding Domain

**DOI:** 10.3390/cells12030427

**Published:** 2023-01-27

**Authors:** Benan Temizci, Seren Kucukvardar, Arzu Karabay

**Affiliations:** 1 Molecular Biology-Genetics and Biotechnology, Graduate School, Istanbul Technical University, 34469 Istanbul, Turkey; 2 Department of Molecular Biology and Genetics, Istanbul Technical University, 34469 Istanbul, Turkey

**Keywords:** Spastin, Pin1, phosphorylation, migration, invasion, glioblastoma

## Abstract

Microtubule-severing protein Spastin has been shown to co-localize with actin in migratory glioblastoma cells and is linked to glioblastomas’ migration and invasion capacity. However, the effectiveness of Spastin in glioblastoma migration and the molecular mechanism underpinning the orientation of Spastin towards actin filaments remain unknown. Here, we demonstrated that Spastin plays an active role in glioblastoma migration by showing a reduced migratory potential of T98G glioblastoma cells using real-time cell analysis (RTCA) in Spastin-depleted cells. Pull-down assays revealed that a cis–trans isomerase Pin1 interacts with Spastin through binding to the phosphorylated Pin1 recognition motifs in the microtubule-binding domain (MBD), and immunocytochemistry analysis showed that interaction with Pin1 directs Spastin to actin filaments in extended cell regions. Consequently, by utilizing RTCA, we proved that the migration and invasion capacity of T98G glioblastoma cells significantly increased with the overexpression of Spastin, of which the Pin1 recognition motifs in MBD are constitutively phosphorylated, while the overexpression of phospho-mutant form did not have a significant effect on migration and invasion of T98G glioblastoma cells. These findings demonstrate that Pin1 is a novel interaction partner of Spastin, and their interaction drives Spastin to actin filaments, allowing Spastin to contribute to the glioblastomas’ migration and invasion abilities.

## 1. Introduction

Glioblastoma, also designated as Isocitrate Dehydrogenase wild-type (IDH-wt) glioma in the World Health Organization (WHO) Classification of Central Nervous System (CNS) Tumors in 2021, is the most frequent malignant adult brain tumor and is classified as CNS WHO grade 4 [1]. In addition to exhibiting rapid cellular growth, the most important characteristic of glioblastoma cells is the ability to easily infiltrate and invade the surrounding healthy brain tissue, resulting in high recurrence and death rates [2].

Invasive cancer cells use their innate migratory capacity and form specialized protrusions termed invadopodia to invade adjacent tissues [3]. Although the driving force for the formation of invadopodia is provided by the polymerization of actin filaments lying below the cell cortex, microtubules are needed for subsequent elongation of protrusions [4]. The formation of these specialized protrusions, which equip tumor cells with the ability to invade, is comparable to the formation of axonal branching in neurons. Neuronal axonal branching is provided by microtubule-severing proteins that cut microtubules into small pieces at branch sites to promote arborization [5]. Although the role of microtubule-severing proteins in neurons is well understood, their involvement in cancer cell migration and invasion has yet to be elucidated.

Microtubule-severing protein Spastin is a member of the AAA (ATPases Associated with diverse cellular Activities) protein family and cuts microtubules in an ATP-dependent manner [6]. Spastin is classified into two main isoforms, each of which is coded from a different translational initiation site. Translation from the first ATG results in the full-length protein consisting of 616 amino acids (~68 kDa), whereas translation from the second ATG results in the short form consisting of 530 amino acids (~58 kDa) that lacks the first 86 residues containing hydrophobic region in the N-terminal of the full-length Spastin [7]. Except for the hydrophobic region, both isoforms have identical sequence and domains: The N-terminal region of each Spastin isoform contains the microtubule-interacting and trafficking (MIT) domain, which is responsible for interaction and intracellular trafficking, while the C-terminal portion of the Spastin is the AAA domain, which catalyzes the hydrolysis of ATP required to cut microtubules. Moreover, the microtubule-binding domain (MBD) comprises amino acids from 270 to 328, which are necessary for the binding of Spastin to microtubules to cut [8,9,10]. Aside from the fact that both Spastin isoforms are responsible for microtubule severing, their intracellular locations and hence cellular activities differ. While the hydrophobic region at the N-terminus of the full-length Spastin isoform (also known as M1) permits it to localize to the endoplasmic reticulum, the short isoform (known as M87) lacking this region is distributed in the cytoplasm [11]. Since pathogenic mutations in the SPG4 gene encoding Spastin lead to the development of hereditary spastic paraplegia (HSP), earlier investigations on Spastin function have mostly focused on functions of M1 isoform which is thought to be responsible for HSP in neuronal cells [11,12,13]. However, the function of M87-Spastin in dividing cells needs to be investigated further since, unlike M1-Spastin, which is prevalent in the spinal cord and expressed at a low level in other tissues and cell lines, M87-Spastin is expressed ubiquitously [12]. Spastin has recently been linked to glioblastoma cell motility, as it was shown that the level of M87-Spastin increased in correlation with glioblastoma cell invasion capability, and Spastin was found to be co-localized with actins in migratory glioblastoma cells [14]. Nevertheless, the molecular mechanism underpinning Spastin’s motion from microtubules to actin filaments, as well as its influence on cell migration, has yet to be uncovered.

During cell migration, serine/threonine kinase-dependent signaling pathways may transiently shift the subcellular localization of migration regulatory proteins [15]. Pin1, a peptidyl-prolyl cis–trans isomerase enzyme, recognizes phosphorylated Ser/Thr-Pro (p-Ser/Thr-Pro) motifs in its target proteins and regulates their functions by causing changes in their structures [16,17,18]. Previously, we determined that Pin1, whose expression also increases in correlation with the tumor grade of gliomas [19], contributed to tumorigenic properties of gliomas by playing an active role in cell growth, migration, and angiogenic potential [20]. Since Pin1 is known to alter the subcellular localization of its target proteins [21], in this study, we investigated whether Pin1 plays a role in the transient localization change in Spastin in migrating T98G glioblastoma cells. Here, we identified Pin1 as a novel interaction partner of Spastin and showed that this interaction is required for transient co-localization of Spastin with actin filaments to enhance migration and invasion of T98G glioblastoma cells.

## 2. Materials and Methods

### 2.1. Vector Construction

**Figure 1:**
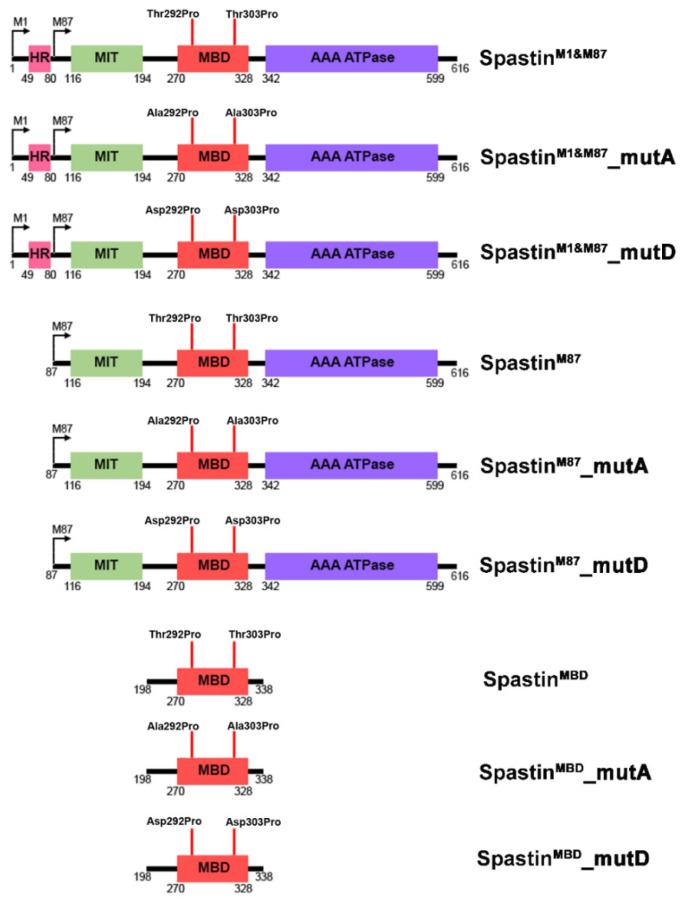
The representative illustrations of the cloned Spastin constructs used in the study. The full-length wild-type or mutant Spastin constructs expressing both M1 and M87-Spastin isoforms are named Spastin^M1&M87^ (Thr292Pro&Thr303Pro), Spastin^M1&M87^_mutA (Ala292Pro&Ala303Pro), or Spastin^M1&M87^_mutD (Asp292Pro & Asp303Pro), and wild-type or mutant Spastin constructs expressing only the M87 isoform of Spastin are named Spastin^M87^, Spastin^M87^_mutA, and Spastin^M87^_mutD, respectively. In addition, the wild-type or mutant constructs expressing only the microtubule-binding domain (MBD) of Spastin consisting of amino acids from 198 to 338 are named Spastin^MBD^, Spastin^MBD^_mutA, and Spastin^MBD^_mutD.

The Pin1-Flag vector was a kind gift from Dr. Aslı Kumbasar. All Spastin vectors designed to be used in this study are shown schematically in Figure 1. The Spastin^M1&M87^ vector expressing both M1 and M87 isoforms due to the presence of CCACC Kozak sequence at the beginning of the sequence was constructed by cloning the full-length Spastin-coding sequence into pcDNA3.1 (+)/myc-His A vector (Addgene, Watertown, MA, USA) at HindIII/XhoI restriction sites. Spastin^M1&M87^_mutA (T292A & T303A) and Spastin^M1&M87^_mutD (T292D & T303D) mutants were obtained by site-directed mutagenesis in the Spastin^M1&M87^ vector using a Q5 Site-Directed Mutagenesis Kit (New England Biolabs, Ipswich, MA, USA) and analyzed by Sanger sequencing. Spastin^MBD^ vectors (Spastin^MBD^, Spastin^MBD^_mutA, and Spastin^MBD^_mutD) containing only the MBD domain (198-338 aa) of Spastin and M87-Spastin vectors (Spastin^M87^, Spastin^M87^_mutA, and Spastin^M87^_mutD) expressing only the M87 isoform of Spastin (87-617 aa) were constructed by cloning the DNA fragments amplified from wild-type and mutant full-length Spastin vectors into pcDNA3.1 (+)/myc-His A vector (Addgene, Watertown, MA, USA) at HindIII/XhoI restriction sites. The primers used in polymerase chain reactions (PCR) performed for the amplification of DNA fragments or site-directed mutagenesis are listed in Table S1.

### 2.2. Cell Culture, Transfection and Treatment

T98G glioblastoma cells and HEK293T embryonic kidney cells were cultured in a 37 °C CO_2_ incubator in Dulbecco’s modified Eagle’s medium (DMEM) (Gibco, Life Technologies, Carlsbad, CA, USA) supplemented with 10% fetal bovine serum (Gibco, Life Technologies, Carlsbad, CA, USA). For pull-down assays, HEK293T cells were transfected with 5 μg of each Spastin vector and/or 4 μg of Pin1-Flag vector using polyethylenimine (PEI) in a 1:3 ratio. Moreover, 50 ng/mL epidermal growth factor (EGF) (Gibco, Life Technologies, Carlsbad, CA, USA) was applied to T98G cells for 5 h. For immunocytochemistry (ICC) analysis, T98G cells were transfected with 1.5 μg of each Spastin vector using PEI in a 1:4 ratio. Separately, T98G cells were treated with 50 ng/mL EGF for 24 h or treated with 10 µM paclitaxel (Sigma-Aldrich, St. Louis, MO, USA) for 18 h. For real-time cell migration analysis, T98G cells were transfected with 50 nM non-targeting (NT) (GE Healthcare Dharmacon, Lafayette, CO, USA) or Spastin siRNAs (GE Healthcare Dharmacon, Lafayette, CO, USA) using DharmaFECT 2 transfection reagent (GE Healthcare Dharmacon, Lafayette, CO, USA) for 48 h. T98G cells were also transfected with 5 µg mock or each mutant M87-Spastin vector using PEI in a 1:4 ratio for real-time cell migration and invasion analysis.

### 2.3. Antibodies

Pin1 antibody (#sc-46660, Santa Cruz Biotechnology, Dallas, TX, USA) was used for immunoprecipitation (IP) of endogenous Pin1. The following antibodies were used for immunoblotting: Pin1 (#sc-46660, Santa Cruz Biotechnology, Dallas, TX, USA); Spastin (#PA5-44807, Invitrogen, Carlsbad, CA, USA); Myc-tag (#2276S, Cell Signaling Technology, Danvers, MA, USA); Myc-tag (#2278S, Cell Signaling Technology, Danvers, MA, USA); Flag-tag (#F7425-2MG, Sigma-Aldrich, St. Louis, MO, USA); β-actin (#8457S, Cell Signaling Technology, Danvers, MA, USA); β-actin (#3700S, Cell Signaling Technology, Danvers, MA, USA); Anti-mouse IgG VeriBlot for IP secondary antibody (ab131368, Abcam, Cambridge, UK); Anti-mouse IgG HRP-linked antibody (#7076S, Cell Signaling Technology, Danvers, MA, USA); IRDye 800CW Goat Anti-Mouse (#5257S, Cell Signaling Technology, Danvers, MA, USA); and IRDye 680RD Goat Anti-Rabbit (#5366S, Cell Signaling Technology, Danvers, MA, USA). Antibodies and dyes used in ICC experiments are as follows: Spastin (#H00006683-M02, Novus Biologicals, Centennial, CO, USA); ꞵ-tubulin (#ab6160, Abcam, Cambridge, UK); Myc-tag (#2276S, Cell Signaling Technology, Danvers, MA, USA); Alexa Fluor® 647 Conjugate Anti-rat IgG (#4418, Cell Signaling Technology, Danvers, MA, USA); Alexa Fluor® 594 Conjugate Anti-mouse IgG (#8890, Cell Signaling Technology, Danvers, MA, USA); and phallacidin (#B607, Invitrogen, Carlsbad, CA, USA).

### 2.4. Immunoprecipitation and Pull-Down Assays

For the endogenous Spastin–Pin1 interaction analysis, T98G cells were lysed in IP-lysis buffer (50 mM Tris-HCl, pH 7.6; 150 mM NaCl; 0.5 M EDTA, 1% NP-40, and protease inhibitors). T98G cell lysates (750 µg) were immunoprecipitated with 2 µg Pin1 antibody (#sc-46660, Santa Cruz Biotechnology, Dallas, TX, USA) and incubated overnight, at 4 °C. The samples were then rotated with Dynabeads^TM^ Protein G (Invitrogen, Carlsbad, CA, USA), at 4 °C, for 4 h. After the incubation, the samples were placed on a magnetic platform, and the supernatant was removed. After magnetic beads were washed three times with IP-lysis buffer, the beads were boiled in sodium dodecyl sulfate (SDS)-loading buffer. 

For the analysis of Spastin–Pin1 interaction based on the mutations in Pin1 recognition motifs in the MBD region of Spastin, 700 µg lysates from Spastin and/or Pin1-overexpressed HEK293T or T98G cells were incubated with 30 µL MagneHis^TM^ Ni Particles (Promega, Madison, WI, USA), at 4 °C, for 2.5 h. After the incubation, magnetic beads were washed three times with washing/binding buffer. Then, nickel particles were resuspended in 30 µL MagneHis elution buffer. Finally, to investigate the co-precipitation of Pin1 with either endogenous or exogenous Spastin, Western blotting (WB) was used to separate precipitated protein samples and 3% input samples used as an expression control. WB was performed as described in the immunoblotting section.

### 2.5. Immunoblotting

Protein samples were resolved by SDS-polyacrylamide gel electrophoresis and transferred onto a nitrocellulose membrane (Santa Cruz Biotechnology, Dallas, TX, USA). Membranes were blocked with 5% non-fat dry milk in TBS-T at room temperature (RT) for 1 h. Then, the membranes were incubated with the specified primary antibodies, at 4°C, overnight, and with proper secondary antibodies, at RT, for 1 h. After the incubation with each primary and secondary antibody, the membranes were washed three times with TBS-T. Then, the visualization was performed using the ChemiDoc XRS Imaging System (BioRad, Hercules, CA, USA) or the Licor Odyssey CLx Near-Infrared Fluorescence Imaging System. Densitometric analyses were performed using ImageLab (Version 6.1), Image Studio (Version 5.2) and Adobe Photoshop CS6 software.

### 2.6. Immunocytochemistry

Immunocytochemistry (ICC) analysis was performed by using two alternative fixation protocols, including either paraformaldehyde (PFA) only or glutaraldehyde and PFA. For fixation with PFA protocol, T98G cells were fixed with 4% PFA at RT for 15 min. Then, cells were washed with 1X phosphate-buffered saline (PBS) for 5 min. Fixation using mixed-aldehyde protocol was carried out as follows: T98G cells were fixed with mixed-aldehyde fixation buffer [4% PFA, 0.2% glutaraldehyde, 1X PHEM (PIPES, HEPES, EGTA, MgCl2) and 0.1% Triton] at RT for 15 min. Then, cells were washed with 1X PHEM and PBS for 5 min and then permeabilized with 0.25% Triton-X-100 in 1X PBS at RT for 10 min. After washing twice with 1X PBS, cells were treated with 1% sodium borohydride in 1X PBS. After the washing steps, all samples were blocked with blocking buffer (3% (*w/v*) bovine serum albumin and 0.1% Triton X-100 in PBS) for 1 h at RT. Then, cells were incubated with specified primary antibodies, at 4 °C, overnight. The next day, samples were washed with 1X PBS and treated with secondary antibodies and phallacidin at RT for 1 h. Samples were then washed three times with 1X PBS. ProLong™ Diamond Antifade Mounting Medium (Invitrogen, Carlsbad, CA, USA) was added onto slides, and coverslips were placed onto samples. Cells were visualized by either TCS SP2 SE Confocal Microscope (Leica, Germany) using a 63× oil immersion objective or Leica TCS SP8 MP Confocal Microscope (Leica, Wetzlar, Hessen, Germany) using a 40× objective. All images were captured in a single focal plane using Leica Application Suite X (LAS X) Version 3.5.1 software and then processed with Adobe Photoshop CS6 Version 21.0.2 software.

### 2.7. Cell Migration and Invasion Analysis

XCELLigence Real-Time Cell Analysis (RTCA) DP system (Roche, Basel, Switzerland) was used to analyze cell migration and invasion. For the migration analysis, the lower chamber of a CIM-Plate16 (Agilent Technologies, Santa Clara, CA, USA ) was filled with 160 µL of DMEM (10% FBS, 1% Pen/Strep), and then the upper chamber of the dishes was placed on the lower chamber and incubated, at 37 °C, in 5% CO_2_ for 1 h. After the incubation period, each upper chamber was filled with 50 µL of DMEM containing 3% FBS, and it was taken to be the blank. Spastin siRNA-treated or mutant Spastin^M87^_mutA or Spastin^M87^_mutD-overexpressed T98G cells were trypsinized and resuspended in DMEM containing 3% FBS. Then, 4 × 10^4^ cells were seeded in each well on the upper chamber, and the CIM-Plate16 was maintained in an incubator for another 30 min to allow cell attachment. The xCELLigence system automatically monitored the impedance value of each well every 15 min for 48 h, and a CI value was obtained. Before the real-time invasion analysis, the top chamber of a CIM-Plate16 was coated with 15% Matrigel Basement Membrane Matrix (Corning Incorporated, Corning, NY, USA) in a CO_2_ incubator for 4 h. Then, the procedures applied during the real-time migration analysis were repeated.

### 2.8. Statistical Analysis

GraphPad Prism 8.0.2 software (GraphPad Software Inc., CA, USA) was used for the statistical analyses. The two-tailed Student’s t-test was used to evaluate the difference between the means of two groups. One-way ANOVA was utilized for multiple group comparisons. Error bars in the graphs were generated using ± standard deviations (SD). Statistical significance was set at *p* < 0.05.

## 3. Results

### 3.1. T98G Glioblastoma Cell Migration Capacity Is Reduced in Spastin-Depleted Cells

**Figure 2:**
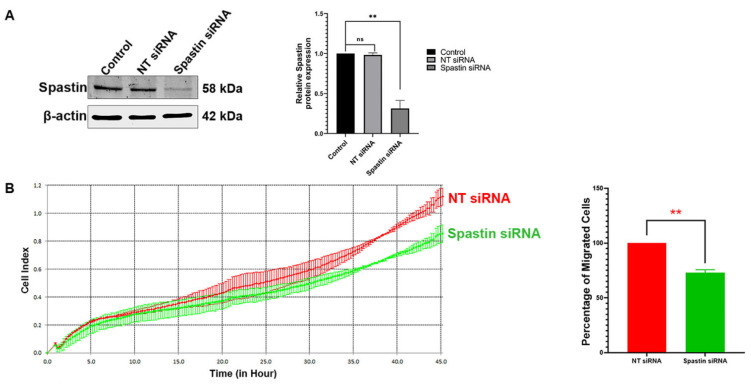
Real-time monitoring and measurement of T98G cell motility upon Spastin depletion. (**A**) Depletion of Spastin expression upon siRNA treatment was analyzed by Western blot (WB). T98G cells were transfected with either Spastin-specific or non-target siRNA for 48 h. Then, Spastin expression was detected with WB using a specific antibody to Spastin. Representative WB is shown and relative densitometric values of Spastin/β-actin are reported in the right panel. Molecular weights of the proteins are reported in kilodalton (kDa). (**B**) xCelligence impedance-based system was used for the migration analysis. Representative result is shown in the left panel. Results were statistically analyzed by t-test from two independent biological replicates containing three independent technical replicates. Data are represented as mean ± SD. n.s. *p* > 0.05, and ** *p* < 0.01 (right panel).

Since earlier research suggested that increased Spastin expression in glioblastomas may be associated with tumor cell motility [14], we initially evaluated the significance of the endogenous Spastin in the migration of T98G cells, an IDH-wt cell line [22] with the highest expression of Spastin among the glioblastoma cells studied [14]. For this aim, we applied real-time cell migration analysis upon acute Spastin depletion achieved by siRNA treatment (Figure 2A). We observed that the index value showing the signal of T98G cells’ transition from the upper well to the lower well was reduced in cells treated with Spastin siRNA (72.9%), indicating a slowing down in migration compared with the control cells treated with NT siRNA (100%) (Figure 2B). These findings demonstrate that Spastin affects migratory capability of T98G cells.

### 3.2. Spastin Is Directed to Actin Filaments in Migration-Induced T98G Glioblastoma Cells

**Figure 3:**
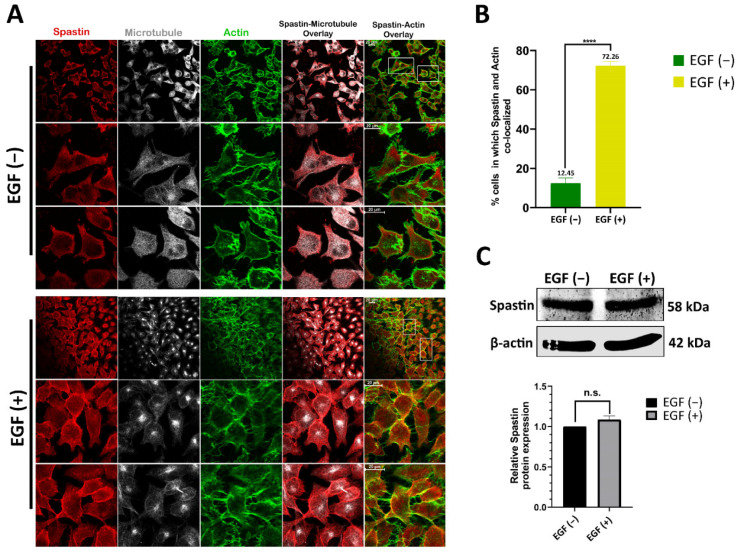
Spastin is directed to actin filaments upon stimulation with EGF. (**A**) The co-localization of Spastin with actin upon EGF treatment was analyzed by immunocytochemistry (ICC) analysis. T98G cells were treated with EGF for 24 h, and non-treated cells were used as controls. For the ICC analysis, cells were fixed with mixed-aldehyde and stained with Spastin antibody (red), microtubule antibody (gray), and phallacidin (green). The lower two panels for each section show higher magnification views of the boxed areas indicated in the upper panels. (**B**) Bar graphs show the percentage of cells possessing Spastin co-localization with actin filaments in control and EGF-treated conditions. The quantification was performed by dividing the number of EGF-treated cells containing Spastin co-localization with actin to the number of untreated control cells containing Spastin co-localization with actin. Results were statistically analyzed by unpaired t-test (*n* > 150) and data are represented as mean ± SD; **** *p* < 0.0001. (**C**) Spastin expression was detected with Western blot (WB) using a specific antibody to Spastin. Representative WB is shown, and relative densitometric values of Spastin/β-actin are reported in the lower panel. Molecular weights of the proteins are reported in kilodalton (kDa). Data are represented as mean ± SD. n.s. *p* > 0.05.

Cell migration involves cytoskeleton remodeling, and while the arrangement of actin filaments is known to be a driving factor for migration, it is also known that microtubules play a role in the elongation of the extended cell regions involved in migration [4]. To further analyze the role of Spastin in migration, we also examined the localization of endogenous Spastin in migration-induced and control T98G cells. EGF is known to promote cell motility in glioblastoma, like in many other cancers [23,24]. Thus, we investigated Spastin localization in response to EGF treatment, and ICC analysis revealed that the localization of endogenous Spastin in EGF-treated cells at the cell periphery where actin filaments were localized was significantly increased compared to control cells that were not stimulated by EGF (Figure 3A,B). Moreover, we found that there was no significant change in Spastin expression depending on EGF treatment (Figure 3C). These findings indicate that Spastin, which is known to co-localize with microtubules for its function, is enriched at the periphery or extended regions of migratory T98G cells upon EGF treatment, despite the fact that its expression is not increased.

### 3.3. Spastin Orientation to Actin Filaments Is Controlled by Its Interaction with PIN1

**Figure 4:**
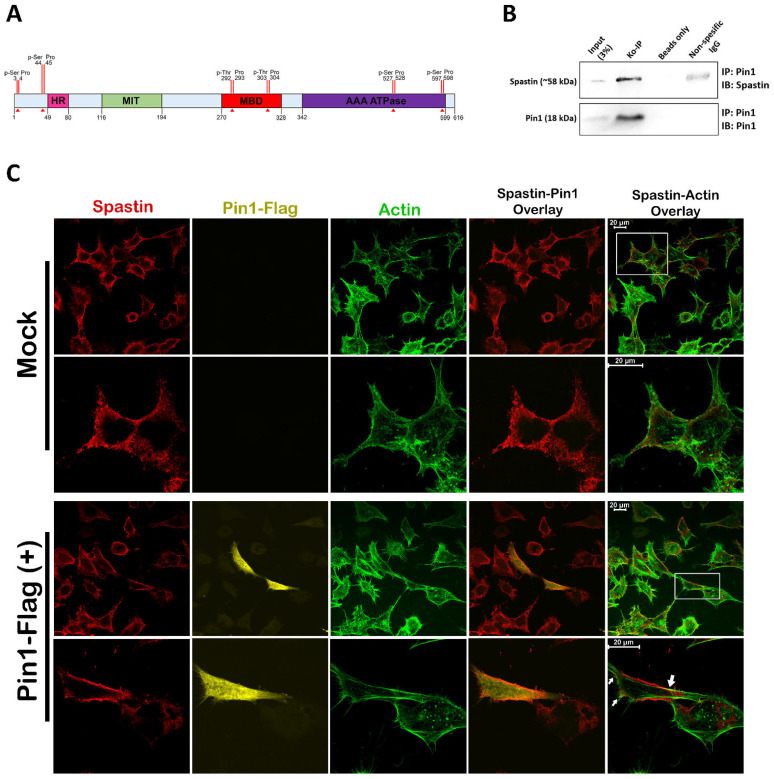
Spastin interacts with Pin1. (**A**) Schematic illustration of the Pin1 recognition motifs on Spastin. HR: hydrophobic region; MIT: microtubule-interacting and -trafficking; MBD: microtubule-binding domain; AAA: ATPases associated with diverse cellular activities. Red arrows indicate potential phosphorylation sites of Pin1 recognition motifs on Spastin. (**B**) Co-immunoprecipitation assay was used to investigate the interaction between endogenous Spastin and Pin1. T98G cell lysates were used in immunoprecipitation with anti-Pin1 antibody, while normal mouse IgG and only beads were used as negative controls. Spastin and Pin1 were detected by immunoblotting with the indicated antibodies. (**C**) The alteration of endogenous Spastin localization mediated by Pin1-Flag protein overexpression was determined by immunocytochemistry analysis. T98G cells were transfected with either mock or Pin1-Flag vectors for 24 h. Then, cells were fixed with PFA and stained with Spastin antibody (red), Flag-tag antibody (yellow), and phallacidin (green). The lower panel for each section shows higher magnification views of the boxed areas indicated in the upper panels and arrows indicate color transformation caused by the co-localization of Spastin with actin filaments driven by Pin1 overexpression.

Post-translational modifications such as phosphorylation control the temporary localization change in proteins involved in the regulation of cellular processes such as migration [15]. Moreover, earlier research has shown that EGF receptor (EGFR) activation through EGF stimulation causes global phosphorylation of many cellular proteins [25]. Given that EGF did not influence the expression level of Spastin (Figure 3C) and based on the literature [15,25], we hypothesized that the increase in Spastin co-localization with actin filaments (Figure 3A,B) could be caused by EGF-mediated Spastin phosphorylation, which might steer Spastin’s direction to actin filaments during cell migration. Thus, we investigated possible phosphorylation sites of Spastin by bioinformatic analyses and identified six different Pin1 recognition motifs (Ser/Thr-Pro motifs), including two at the N-terminal, two at the C-terminal, and two at the MBD regions of Spastin (Figure 4A). Thereupon, we used co-immunoprecipitation (co-IP) analysis to examine the potential Spastin–Pin1 interaction and observed that Spastin co-precipitated with Pin1 in T98G cell lysates (Figure 4B). Following that, we performed ICC analysis to reveal whether the interaction of Spastin with Pin1 affects the cellular distribution of Spastin. We observed that upon Pin1 overexpression, Spastin was enriched at the cell periphery where actin filaments were abundant, whereas it was dispersed in the cytosol in control cells (Figure 4C). These results point out that the interaction of Spastin with Pin1 may play an active role in directing the localization of Spastin with actin filaments.

**Figure 5:**
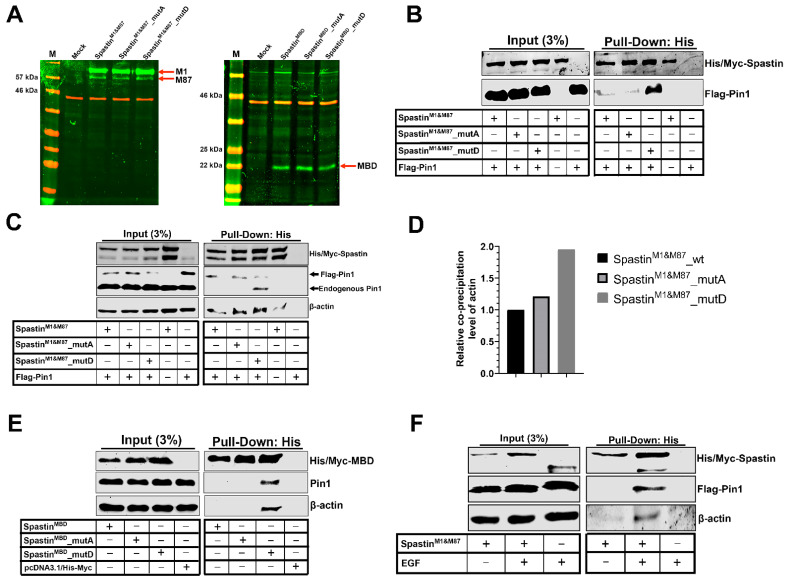
Pin1 interacts with Spastin through Pin1 recognition motifs in its microtubule-binding domain (MBD). (**A**) The expression levels of the proteins expressed from either the full-length or MBD-only Spastin constructs were analyzed through Western blot (WB). Green bands indicate the expressions of either the full-length Spastin (left panel) or MBD-only proteins (right panel) in the wild-type or mutant forms. ꞵ-Actin observed as red bands was used as the loading control. (**B**,**C**) The pull-down assay was used to examine the interaction of the full-length Spastin with Pin1 (**B**) and actin (**C**), respectively. Spastin^M1&M87^ proteins were precipitated from cell lysates (HEK293T in **B**, and T98G in **C**) overexpressing the wild-type or mutant Spastin^M1&M87^ proteins dually with Pin1-Flag. Then, co-precipitation of Pin1 or actin with each wild-type and mutant Spastin proteins was investigated by WB. (**D**) The bar graphs represent the amount of co-precipitated actin with the full-length wild-type or mutant Spastin^M1&M87^ protein. The quantification was performed by dividing the amount of precipitated actin to the amount of precipitated wild-type or mutant Spastin proteins. (**E**) The interaction between Spastin’s MBD region with Pin1 was investigated by pull-down assay. Each Spastin^MBD^ protein was precipitated from HEK293T cells overexpressing either the wild-type or mutant Spastin^MBD^ constructs singly. Then, co-precipitation of the endogenous Pin1 or actin with each wild-type or mutant Spastin protein was investigated by WB. (**F**) To analyze the impact of EGF treatment in the interaction of Spastin with Pin1, pull-down experiment was performed. Exogenous wild-type Spastin protein was precipitated from lysates isolated from Spastin^M1&M87^-overexpressed T98G cells either treated with EGF or not. The co-precipitation of Flag-Pin1 or actin with Spastin was investigated by WB.

Since the MBD is the primary microtubule-binding site of Spastin [26], we hypothesized that the Ser/Thr-Pro motifs in the MBD region of Spastin may play an active role in gearing Spastin’s localization between microtubules and actins. Therefore, we generated wild-type Spastin^M1&M87^ (Thr292Pro&Thr303Pro) and mutant full-length Spastin constructs, including Spastin^M1&M87^_mutA with Thr residues converted to Ala (Ala292Pro&Ala303Pro; non-phosphorylatable mutant) and Spastin^M1&M87^_mutD with Thr residues converted to Asp (Asp292Pro&Asp303Pro; phospho-mimetic mutant) (Figure 1). We initially used both M1 and M87 Spastin isoforms expressing constructs (Figure 5A, left panel) to investigate Spastin–Pin1 interaction. Pull-down analysis with the wild-type and mutant Spastin-expressing HEK293T cells revealed that only Spastin^M1&M87^_mutD protein was co-precipitated with Pin1 (Figure 5B). We also performed the pull-down analysis with the wild-type and mutant Spastin constructs expressing T98G glioblastoma cells to confirm the involvement of Pin1 in the temporary alteration of Spastin’s localization in T98G cells (Figure 4C). We showed that the level of co-precipitated actin with Spastin is increased with the phospho-mimetic Spastin, Spastin^M1&M87^_mutD, which binds precisely and strongly to the endogenous Pin1 (Figure 5C,D). In addition, to further understand the importance of Pin1 recognition motifs (292Thr-Pro and 303Thr-Pro) in the MBD region (Figure 4A) for Spastin’s orientation to actins, we performed co-precipitation analysis with constructs designed for expressing either the wild-type Spastin^MBD^ or the mutant Spastin^MBD^_mutA and Spastin^MBD^_mutD proteins comprising only the MBD region of Spastin (Figure 1). Pull-down experiments using these constructs expressing ~22 kDa protein (Figure 5A, right panel) showed that only Spastin^MBD^_mutD protein co-precipitated with the endogenous Pin1 and actin (Figure 5E). These findings indicate that Spastin interacts with Pin1 upon phosphorylation of Pin1 recognition motifs in its MBD region and only interacts with actins when sustained phosphorylation of these motifs is imitated (Figure 5E). Upon observing these results, we investigated the reason why Pin1 did not co-precipitate with either the wild-type full-length Spastin (Spastin^M1&M87^) or the wild-type Spastin^MBD^ proteins, as opposed to the mutant Spastin^M1&M87^_mutD and Spastin^MBD^_mutD proteins (Figure 5B,C,E). In order to understand if inadequate phosphorylation of the overexpressed Spastin protein in the cells was responsible for the lack of this interaction, we performed the pull-down assay by triggering the phosphorylation of the exogenously overexpressed Spastin and investigated the interaction of exogenous Spastin^M1&M87^ with Pin1 upon EGF treatment based on the observed effect of EGF administration on the localization of Spastin in T98G glioblastoma cells (Figure 3). We showed that the Flag-tagged Pin1 co-precipitated with the overexpressed wild-type Spastin^M1&M87^ only when cells were treated with EGF (Figure 5F). Pin1 recognition motifs on Spastin’s MBD region can be phosphorylated by EGF-triggered kinases such as cyclin-dependent kinases (CDKs), mitogen-activated protein kinases (MAPKs), protein kinase C (PKC), protein kinase A (PKA), Glycogen synthase kinase 3 (GSK3), or casein kinases (CK1 and CK2) (S1 Spreadsheet) [25,27]. The fact that Pin1 co-precipitated with Spastin^M1&M87^ only when EGF was applied indicated that the kinases triggered by EGF may provide adequate phosphorylation of Pin1 recognition motifs present in the MBD region of the Spastin.

### 3.4. Only the M87-Spastin Isoform Is Directed to Actin Filaments Due to Its Interaction with Pin1

**Figure 6:**
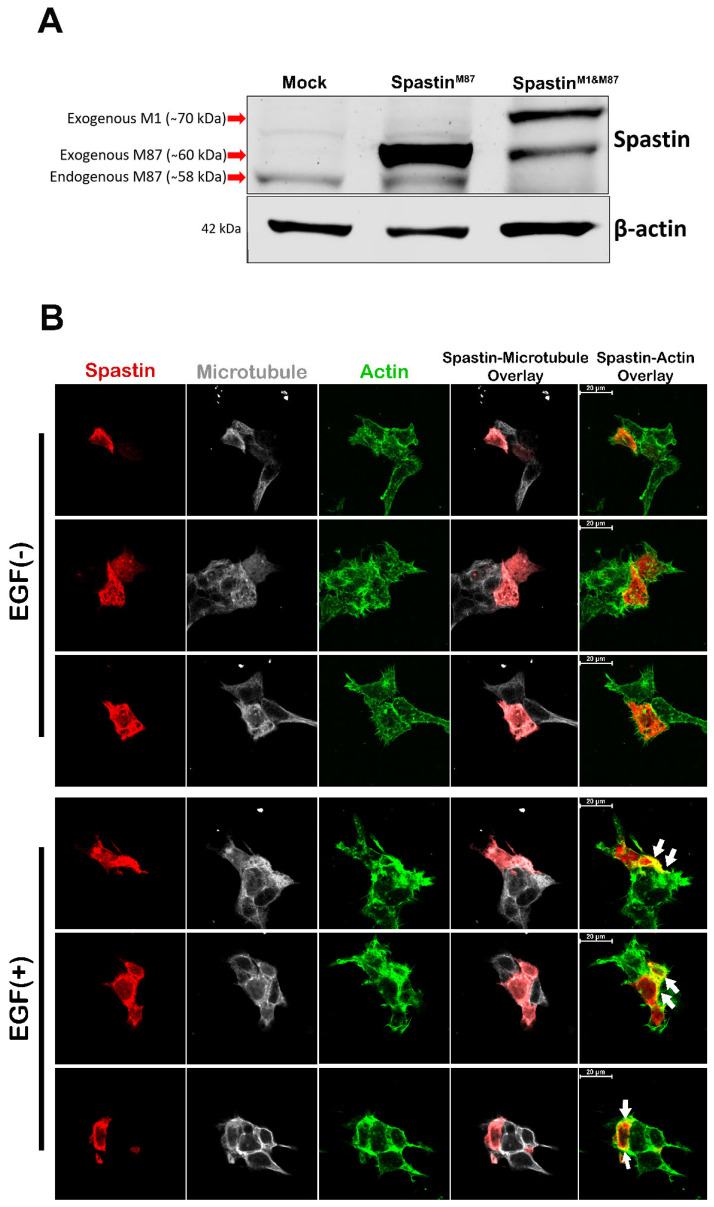

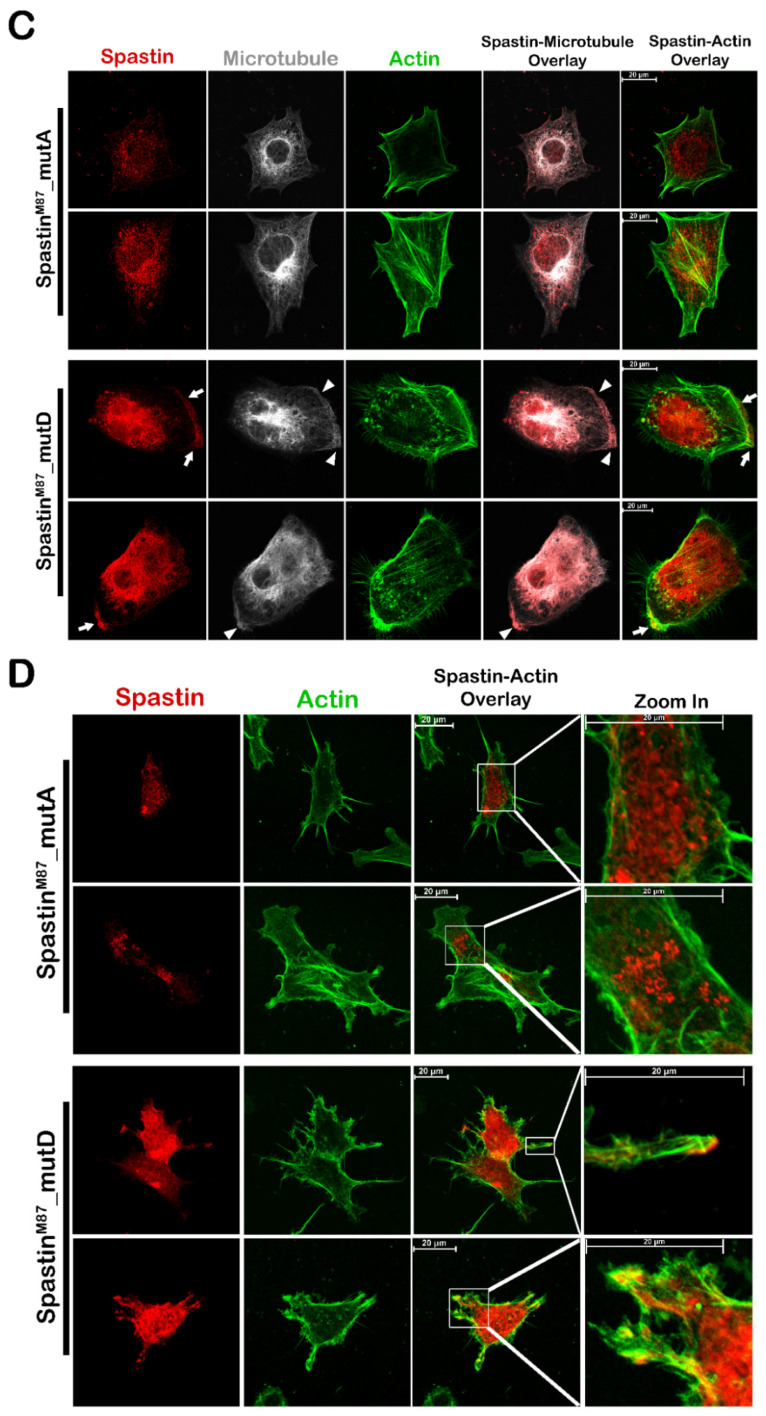
M87-Spastin is directed to actin filaments through phosphorylation of the microtubule-binding domain (MBD). (**A**) The expression levels and molecular weights of the proteins expressed from Spastin^M87^, or Spastin^M1&M87^ vectors were analyzed through Western blot (WB). T98G cells were transfected with either mock, Spastin^M87^, or Spastin^M1&M87^ vectors, and then endogenous or overexpressed Spastin expressions were detected by WB using a Spastin-specific antibody. β-actin was used as the loading control. (**B**) Immunocytochemistry (ICC) analysis was used to examine the impact of EGF treatment on the localization of wild-type M87-Spastin. T98G cells were transfected with Spastin^M87^ vector for 24 h with or without EGF treatment. Then, cells were fixed with PFA and stained with Myc-tag antibody for Spastin (red), phallacidin for actin filaments (green), and tubulin antibody for microtubule (gray). (**C**,**D**) ICC was used to detect the localization of mutant M87 proteins relative to both microtubules and actins (**C**) or relative to actin filaments with only higher magnification (**D**). T98G cells were transfected with either Spastin^M87^_mutA or Spastin^M87^_mutD vectors for 24 h. Then, cells were fixed with PFA and stained with Myc-tag antibody for Spastin (red), phallacidin for actin filaments (green), and tubulin antibody for microtubule (gray). Arrows indicate color transformation caused by the co-localization of Spastin with actin filaments (in **B**,**C**; lane of Spastin actin overlay), while arrowheads indicate microtubule structure that enriched with Spastin and actin filaments (in **C**; lane of microtubule).

Following the determination that the interaction of Spastin with Pin1 was induced by phosphorylation in the MBD region of Spastin, we examined if there was a difference in the localization of Spastin isoforms due to phosphorylation. Since T98G glioblastoma cells mainly express the M87-Spastin isoform (Figure 6A), to analyze the role of M87-Spastin in detail and compare the isoforms, we cloned the wild-type (Spastin^M87^) and mutant (Spastin^M87^_mutA and Spastin^M87^_mutD) M87-Spastin constructs (Figure 1) expressing only the M87-Spastin isoform (Figure S1). We first performed ICC analysis with the wild-type M87-Spastin to examine if there was a difference in the localization of the wild-type Spastin^M87^ due to EGF treatment, and the results revealed that the exogenous wild-type Spastin^M87^ is directed to actin upon EGF treatment (Figure 6B). Next, we also examined the subcellular localization of the Spastin^M87^ mutants and showed that while both Spastin^M87^_mutA and Spastin^M87^_mutD were dispersed in the cells, Spastin^M87^_mutD protein was more accumulated at the cell periphery and in the extended regions of the cells, wherein it was observed to be co-localized with actins, unlike Spastin^M87^_mutA protein (Figure 6C). After discovering that the phospho-mimetic Spastin concentrates in the actin-rich regions of the cells, we sought to examine its co-localization with actin in detail. We observed that the Spastin^M87^_mutD protein was co-localized with the actin in the cell extensions (Figure 6D, lower panel), but the Spastin^M87^_mutA protein was distributed throughout the cell cytoplasm without being directed to the extensions (Figure 6D, upper panel). In addition, we examined the change in the localization of the full-length Spastin^M1&M87^ construct, which expresses more M1-Spastin, upon EGF treatment. ICC results showed that the wild-type Spastin^M1&M87^ was dispersed in the cytoplasm and did not co-localize with actins in either control [EGF (−)] or EGF-treated [EGF (+)] cells (Figure S2). We also comparatively analyzed the intracellular localization of the full-length phospho-mimetic (Spastin^M1&M87^_mutD) and non-phosphorylatable (Spastin^M1&M87^_mutA) proteins, which differ in their interaction with Pin1, and the results showed that neither Spastin^M1&M87^_mutA nor Spastin^M1&M87^_mutD co-localized with actins in T98G cells, as observed with Spastin^M1&M87^ proteins (Figure S3). All these findings suggest that only the M87-Spastin isoform is directed to actins upon phosphorylation and hence upon its interaction with Pin1, while the M1-Spastin isoform does not show orientation to actin filaments.

**Figure 7:**
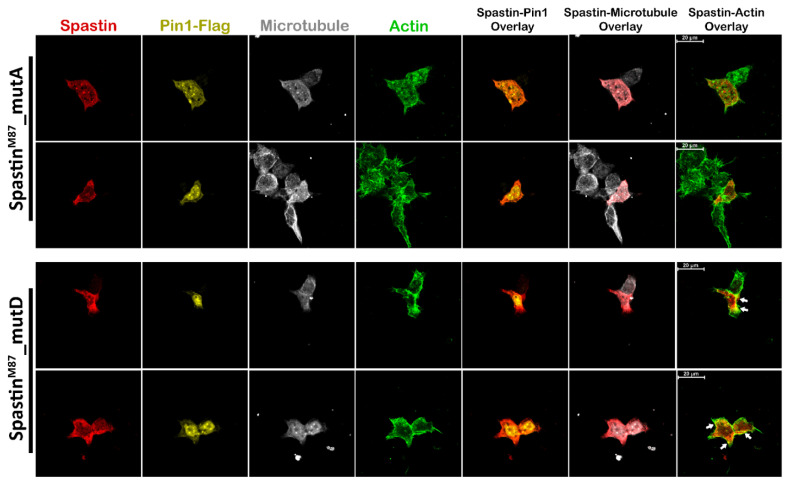
M87-Spastin is directed to actin filaments due to its interaction with Pin1. T98G cells were dually transfected with each mutant M87 vector (Spastin^M87^_mutA or Spastin^M87^_mutD) and Pin1-Flag vector. After 24 h of transfection, cells were fixed with PFA and stained with Myc-tag antibody for Spastin (red), Flag-tag antibody for Pin1 (yellow), phallacidin for actin filaments (green), and tubulin antibody for microtubule (gray). Arrows indicate color transformation caused by the co-localization of Spastin with actin filaments.

After we showed that the phospho-mimetic mutation in the MBD region of Spastin leads to a significant change in the localization of M87-Spastin (Figure 6C,D; lower panels), we investigated the localization of mutant M87 proteins in T98G cells depending on Pin1-Flag overexpression to explain whether Pin1 has a direct effect on the motion of Spastin from the microtubules towards the actin filaments in the cell cortex. The ICC results revealed that the dispersed Spastin^M87^_mutD protein in the cell cytoplasm, when overexpressed alone (Figure 6C,D), was directed to the cell cortex upon Pin1 overexpression, and the co-localization of the Spastin^M87^_mutD protein with actin filaments was increased (Figure 7). On the other hand, despite the overexpression of Pin1, Spastin^M87^_mutA protein remained diffused in the cytoplasm and co-localized with the microtubules as expected (Figure 7). These results demonstrate that the interaction of Spastin with Pin1 through the phosphorylated Pin1 recognition motifs (Thr292Pro and Thr303Pro) on the MBD region plays an active role in the temporary alteration of Spastin’s localization between actins and microtubules.

### 3.5. Microtubule Stabilization Inhibits Spastin’s Localization Transition from Microtubules to Actin Filaments

**Figure 8:**
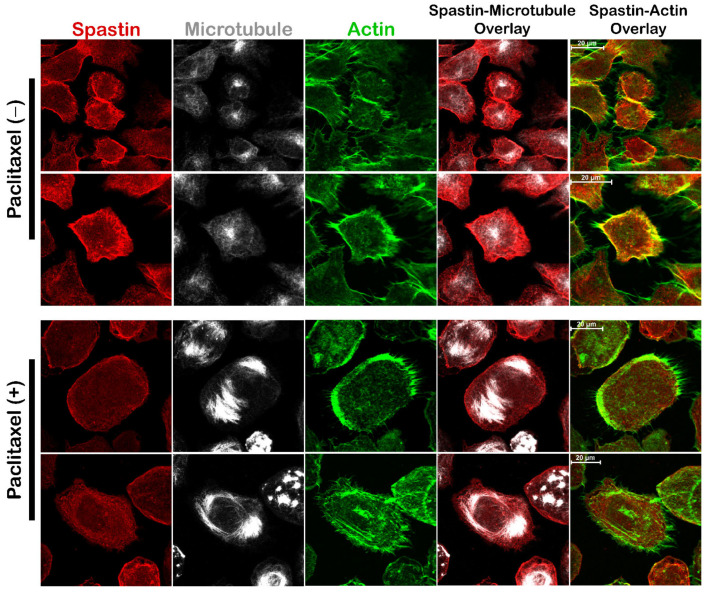
Spastin could not be directed to actin filaments upon microtubule stabilization. T98G cells were treated with EGF for 24 h and with paclitaxel (Paclitaxel (+); lower panel) for 18 h, and the cells which were stimulated with only EGF were used as controls (Paclitaxel (−); upper panel). After treatments, cells were fixed with mixed-aldehyde and stained with Spastin antibody (red), tubulin antibody for microtubule (gray), and phallacidin for actin filaments (green).

After identifying that the interaction between Spastin and Pin1 leads the localization of Spastin from microtubules to actins, we investigated if the re-localization of Spastin is reliant on microtubule dynamics. For this reason, we examined the localization of phospho-mimetic Spastin^M87^__mutD protein in T98G cells upon paclitaxel treatment which reduces microtubule dynamics through stabilizing microtubules [28]. The ICC results showed that overexpressed Spastin^M87^_mutD protein in the paclitaxel-treated cells was co-localized with both microtubule and actin, just as it does in cells where Spastin^M87^_mutD is overexpressed alone (Figure S4). Since paclitaxel treatment is known to be unable to protect the microtubules from severing proteins [29], and accordingly, the overexpressed phospho-mimetic M87-Spastin protein caused extensive severing of microtubules. Therefore, the impact of microtubule stability on Spastin localization could not be truly observable under the effect of overexpressed M87-Spastin protein (Figure S4). Hence, we decided to evaluate the localization of endogenous Spastin after paclitaxel treatment in order to accurately analyze the effect of microtubule stability on the alteration of Spastin’s localization. For this aim, we investigated the localization of endogenous Spastin in T98G cells using the paclitaxel in combination with EGF treatment, which triggers the orientation of Spastin from microtubules to actins. The ICC findings indicated that the co-localization of Spastin with actin, which was detected in cells stimulated with only EGF (Figure 3A, lower panels: EGF (+); Figure 8, upper panels: Paclitaxel (−)) was inhibited in cells where microtubules were stabilized by paclitaxel treatment even in the presence of EGF (Figure 8, lower panels: Paclitaxel (+)). These findings suggested that Spastin needs microtubule dynamicity and presence of short microtubule fragments for its co-localization with actins and enrichment at the cell periphery.

### 3.6. M87-Spastin Triggers Glioblastoma Cell Migration and Invasion Only When Its MBD Region Is Phosphorylated

**Figure 9:**
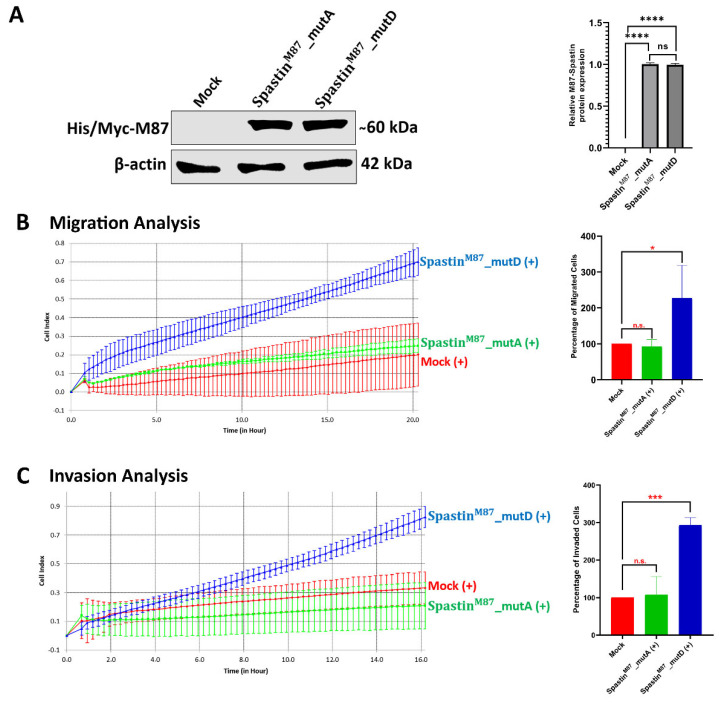
Real-time monitoring and measurement of T98G cell motility upon M87 overexpression. (**A**) Expression levels of exogenous mutant M87-Spastin proteins were analyzed by Western blot (WB). T98G cells were transfected with either mock, Spastin^M87^_mutA, or Spastin^M87^_mutD vectors and analyzed 24 h post-transfection with immunoblotting using Myc-tag antibody for M87 proteins. β-actin was used as a loading control. Representative WB is shown, and relative densitometric values of M87-Spastin/β-actin are reported in the right panel. (**B**,**C**) xCelligence impedance-based system was used for the migration (**B**) and invasion (**C**) analysis. Results were statistically analyzed by one-way ANOVA from two independent biological replicates containing at least three independent technical replicates. Data are represented as mean ± SD; n.s. *p* > 0.05, * *p* <0.05, *** *p* <0.001, and **** *p* < 0.0001 (right panel).

Real-time cell migration analysis was employed to investigate if the orientation of Spastin to actin filaments as a result of its interaction with Pin1 plays an active role in the migration and invasion abilities of T98G glioblastoma cells. For this purpose, both mutant M87 proteins were overexpressed in T98G cells (Figure 9A), and the migration and invasion of these cells were monitored for 48 h and compared with the mock-transfected control cells (Figure 9B,C). During the migration analysis, when the cell index values indicating the signal of T98G cells transitioning from the upper chamber to the lower chamber were examined, it was observed that the index values of the cells overexpressing Spastin^M87^_mutD (227%) increased more rapidly than those of the control cells transfected with mock vector (100%). On the other hand, the index values of the Spastin^M87^_mutA-overexpressed cells (92%) were not significantly different than those of the control cells (Figure 9B). In addition to the migration analysis, we examined whether there was a change in the invasion capacity of T98G cells based on phosphorylation. Similar to the migration analysis results, we found that the index value of Spastin^M87^_mutD-overexpressing cells (287%) increased quite rapidly compared with both control and Spastin^M87^_mutA-overexpressing cells. However, there was not a significant change in Spastin^M87^_mutA-overexpressed cells (119%) compared with the control cells (Figure 9C). All these findings indicate that M87-Spastin could contribute to the migration and invasion ability of T98G glioblastoma cells only if it is directed to actin filaments after interacting with Pin1 owing to its phosphorylation in the MBD region.

## 4. Discussion

Studies on the function of Spastin have been mostly focused on the neuronal axonal transport process, which is dysfunctional in spastic paraplegia type 4 caused by mutations in the *SPG4* gene that produces Spastin [11,12,13]. Investigations in mitotic cells have not proceeded beyond examining the function of Spastin in the ER tubular network; only a study performed by Draberova and colleagues (2011) revealed that Spastin levels are increased in glioblastoma cells interrelatedly with their invasion capacity and that it is co-localized with actin in migratory T98G glioblastoma cells [14], indicating that Spastin might also play a role in cell motility. Although this study implies that Spastin may participate actively in cell migration, there is no evidence in the literature to support this claim.

In this study, we demonstrated that Spastin plays an active role in cell migration by using real-time migration analysis of T98G glioblastoma cells in the presence and absence of Spastin (Figure 2B), establishing for the first time a relationship between Spastin and cell migration. To reveal the molecular mechanism behind Spastin’s function in cell migration, we firstly investigated the intracellular localization of Spastin in T98G cells in which migration was stimulated or not. ICC analysis showed that the co-localization of endogenous Spastin with actin filaments at the periphery or extended regions of the T98G cells was significantly enhanced upon EGF treatment (Figure 3A,B), which triggers the EGFR kinase activity that has been shown to accelerate angiogenesis and invasion of glioblastoma [30,31]. According to studies, EGFR, whose expression is known to increase in association with the severity of glioblastomas [32], is thought to be involved in a signal network within the cell that includes 122 proteins and 211 interactions [33]. Furthermore, Olsen and colleagues (2006) reported that EGF treatment activates EGFR, resulting in the global phosphorylation of 2244 proteins at 6600 sites, with threonine sites accounting for around 12% of these phosphorylation sites [25]. After confirming that the expression level of Spastin was not altered by EGF treatment (Figure 3C), we inquired to identify if it was possible that phosphorylation induces the orientation of Spastin to actin filaments. Therefore, we investigated putative phosphorylation sites in the Spastin sequence, and a bioinformatic analysis showed that Spastin has six different Pin1 recognition motifs, two of which are found in the Spastin’s MBD region (Thr292Pro and Thr303Pro) (Figure 4A), which is primarily responsible for the microtubule binding [26]. Therefore, we inquired to find if there would be an interaction between Spastin and Pin1, whose interaction depends on phosphorylation of the target residues. In addition, co-IP analyses have proved that Pin1 is an interaction partner of Spastin (Figure 4B). Furthermore, we discovered that endogenous Spastin is directed to actins in T98G glioblastoma cells upon Pin1 overexpression, which is comparable to EGF treatment (Figure 4C). Pin1 expression is transcriptionally controlled by E2 transcription factor 1 (E2F1) and has also been shown to be upregulated via E2F1 triggered by H-Ras-oncogenic signaling mediated by EGFR activation [34]. Moreover, Pin1 expression in gliomas is known to increase in correlation with tumor grade, similar to EGFR expression [19]. In accordance with this evidence, our results suggest that since both the phosphorylation of Spastin and the increase in Pin1 expression required for this interaction are induced by EGF treatment, the enhanced EGFR kinase activity in glioblastoma must play a significant role in the orientation of Spastin to actin filaments as a result of its interaction with Pin1.

Pull-down assays using phospho-mimetic or non-phosphorylatable mutants of the Spastin’s MBD demonstrated that phosphorylation of Pin1 recognition motifs in the MBD region mediates interaction between Spastin and Pin1 (Figure 5B,C,E). Nevertheless, which kinases might have a role in the phosphorylation of Spastin at Pin1 recognition motifs located in its MBD region remains to be clarified. To further look into this, we searched for possible kinases that may phosphorylate Thr292 and/or Thr303 residues of Pin1 recognition motifs in the MBD of Spastin using the bioinformatic tools NetPhos 3.1 [35], PPSP [36], and GPS 5.0 [37]. The data obtained by bioinformatic analysis are given in Spreadsheet S1, and our findings led us to propose that the Pin1 recognition motifs located in the MBD region of Spastin might be phosphorylated by Ser/Thr protein kinases that are known to be triggered due to EGFR activation, such as CDKs, MAPKs, PKC, PKA, GSK3, or CKs [25,27]. However, further experimental investigations are needed to determine which specific kinase(s) may have roles in the phosphorylation of MBD and which Thr residues are phosphorylated.

Although we do not exclude possible interactions of Pin1 with Spastin through other putative Pin1 recognition motifs present in Spastin’s N-terminal and AAA-ATPase domains, pull-down (Figure 5E,F) and ICC analyses (Figure 6 and Figure 7) using phospho-mimetic or non-phosphorylatable mutants of the full length or MBD Spastin constructs were able to show that Pin1 interaction through the MBD region of Spastin is required in determining Spastin’s re-localization from microtubules to actins. Although both Spastin^M1&M87^_mutA and Spastin^M1&M87^_mutD proteins were found to co-precipitate with actin (Figure 5C), analyses of Spastin proteins containing only the MBD region proved that only Spastin^MBD^_mutD co-precipitated with actin (Figure 5E). The co-precipitation of the full length Spastin^M1&M87^ or Spastin^M1&M87^_mutA with the actin could be related to the interaction through another Spastin-interacting protein with actin. Pull-down experiments utilizing cell lysates do not necessarily reveal direct interaction with the bait and target proteins, but it will actually show that they are present together as members of a complex [38]. Therefore, we decided that in situ localization analysis of the Spastin is the most accurate method to examine whether it is directed to actin filaments due to its interaction with Pin1 through its MBD region. Therewithal, ICC results revealed that only Spastin^M87^_mutD is enriched at the cell periphery or in protrusions where actins are abundant, whereas Spastin^M87^_mutA is distributed throughout the cell (Figure 6C,D). Similarly, we determined that Spastin^M87^, which could not be adequately phosphorylated due to its overexpression, was broadly disseminated in the cell but only could be directed to actin filaments when its phosphorylation was triggered by EGF (Figure 6A). Altogether, this evidence proved unequivocally that Spastin’s orientation to actin filaments is dependent upon the interaction with Pin1 through Spastin’s MBD region.

Although we discovered that M87-Spastin was directed to actins owing to phosphorylation in its MBD region (Figure 6B,C,D), we were unable to detect co-localization of actin filaments with Spastin^M1&M87^_mutD, which predominantly expresses the M1-Spastin isoform as well as the M87-Spastin isoform (Figure S3). This result indicated that while both Spastin isoforms have the potential to interact with Pin1 due to the presence of MBD in their structures, M87-Spastin could perform specific cellular functions in cell migration and invasion due to its cytoplasmic localization, as opposed to M1-Spastin, which is ER-resident.

On the other hand, ICC analyses investigating the localization of solely overexpressed M87 protein revealed that while some phospho-mimetic Spastin^M87^_mutD protein was found to be oriented towards actins, the majority of the Spastin^M87^_mutD protein was found to be scattered throughout the cytoplasm (Figure 6C,D). In contrast to singly overexpressed M87, when Pin1 protein was overexpressed dually with M87 proteins, Spastin^M87^_mutA protein remained scattered in the cytoplasm, while the majority of Spastin^M87^_mutD protein was enriched at the cell periphery where actin filaments are abundant (Figure 7). These results indicated that the majority of the Spastin^M87^_mutD protein could not be directed to actin when there was an insufficient amount of endogenous Pin1 to interact with the exogenous phospho-mimetic M87-Spastin. Moreover, it is also understood from these results that phosphorylation of Spastin in its MBD is insufficient for its orientation to actin, and that this orientation was only achievable as a result of its interaction with Pin1 and hence its isomerization.

Additionally, we also detected that the microtubule severing activity of both non-phosphorylatable and phospho-mimetic M87-Spastin proteins persist, and their overexpressions result in loss of microtubule mass and disruption of filamentous microtubule structures. While enrichment of short non-filamentous microtubules was observed at the cell periphery where Spastin^M87^_mutD was concentrated (Figure 6C), microtubule mass was diminished without any specific enrichment regions in cells overexpressing Spastin^M87^_mutA (Figure 6C), as was detected in cells overexpressing wild-type Spastin^M87^ (Figure 6B; EGF (-)). Upon observing the presence of enriched short, non-filamentous microtubule structures at the cell periphery with Spastin^M87^_mutD, we questioned if the altered localization of Spastin owing to its interaction with Pin1 was dependent on microtubule dynamics. Therefore, we assessed the impact of paclitaxel, a microtubule stabilizing agent, on the endogenous Spastin localization. ICC analyses demonstrated that when microtubules were stabilized with paclitaxel, endogenous Spastin could not co-localize with actins in the cell periphery, even if EGF was applied (Figure 8). On the other hand, we also observed that in cells treated with paclitaxel, overexpressed Spastin^M87^_mutD protein could still co-localize with actin filaments as paclitaxel treatment was not able to protect microtubule from excessive severing activity of the overexpressed Spastin (Figure S4). These results suggest that Spastin’s co-localization with actin requires not only phosphorylation-dependent Pin1 interaction via its MBD region, but also requires dynamic microtubules and presence of severed short microtubules. Based on this evidence, we believe that Pin1 might act as a mediator of Spastin’s functions. It is tempting to suggest that phosphorylation-dependent Pin1 interaction and hence isomerization of Spastin could facilitate its interaction with motor proteins to carry the cargo microtubules and further process microtubule fragments at the cell periphery to support actin-driven protrusions required for cell migration. A recent study by Kumari and colleagues (2021) identified dynein as a novel interaction partner of Pin1. It is reported in this study that C-terminal domain of the light intermediate chain 1 subunit of dynein (LIC1-CTD) phosphorylation recruits Pin1 to the mitotic dynein complex, and LIC1-CTD phosphorylation regulates mitotic dynein function both directly and through selective Pin1 engagement with a subset of dynein complexes [39]. Likewise, phosphorylation dependent Pin1-Spastin binding could cause conformational change in Spastin through prolyl isomerization, thus resulting in the engagement with adaptors for other functions. The possibility of Pin1-mediated regulation warrants further detailed investigation.

Real-time cell analyses indicating that T98G glioblastoma cell migration or invasion was only induced in cells where Spastin^M87^_mutD protein was overexpressed (Figure 9) imply that Spastin would only induce cell migration or invasion in its phosphorylated form in MBD and, hence, was orientated to actin filaments owing to its interaction with Pin1. Additionally, the decrease in T98G cell migration caused by the silencing of Spastin (Figure 2) indicates that the MBD region of the endogenous Spastin is likely to be present in the phosphorylated form in T98G glioblastoma cells. Spastin, which has been reported to increase in expression in correlation with the invasion capacity of glioblastoma tumor cells, causes severe microtubule loss when overexpressed in dividing cells [12]. Excessive microtubule-severing would normally lead to loss of microtubule cytoskeleton and therefore cell death. However, high proliferation ability [40] and the absence of cellular death [14] of glioblastoma cells in the presence of high levels of Spastin is possibly due to phosphorylation of Spastin because of the increased EGFR activity in glioblastoma cells. As a result of the phosphorylation of Spastin’s MBD region, its interaction with Pin1 prevents excessive microtubule severing and cell death by directing Spastin to the cell periphery. Indeed, Spastin’s orientation to the cell periphery would result in glioblastoma cells having a high migration and invasion ability, causing them to be highly aggressive tumors.

Glioblastoma treatment basically consists of surgical resection, radiation and chemotherapy. However, the most lethal characteristic feature of glioblastomas is their ability to easily invade surrounding healthy brain tissue. As a result, even if a considerable portion of the tumor mass is surgically removed during therapy, due to the high invasion capacity of glioblastoma cells, tumor cells within two centimeters of the resection margin often recur and result in mortality [2]. Moreover, glioblastoma cells with a high migration–invasion capacity are known to be more resistant to therapeutic treatments than proliferative cells [41]. Therefore, it is crucial to identify proteins implicated in the invasion process to improve current glioblastoma therapies. Since our real-time cell analyses clearly show that the interaction between Spastin and Pin1 promotes the migration and invasion capacities, this interaction might be a therapeutic target for glioblastoma treatment. Moreover, because this interaction is unlikely to occur in healthy glial cells due to the low expression of Spastin and Pin1 [14], targeting this interaction may only influence cancer cells; hence, targeting this interaction may be a safe alternative for improving glioblastoma therapy. Given this, developing a bio-mimetic medication that may interfere and disrupt Spastin’s MBD interaction with Pin1 may be a critical step toward treating and preventing the recurrence of glioblastoma tumors.

In conclusion, our findings demonstrated that M87-Spastin has a role in the T98G glioblastoma cell migration and invasion processes, and this involvement is controlled by its shift from microtubules to actin filaments via its interaction with Pin1 through its MBD. Our findings imply that identifying kinases that phosphorylate Pin1 recognition motifs in MBD, will aid in the development of therapeutic drugs that may be beneficial in the treatment of cancer types with high invasion capacity such as glioblastoma.

## Data Availability

This paper and its additional information files contain all of the data obtained or analyzed throughout that study. Other data supporting the findings of this study are accessible from the corresponding authors upon reasonable request.

## Supplementary Materials

The following supporting information can be downloaded at: https://www.mdpi.com/article/10.3390/cells12030427/s1, Table S1: List of primers used for the creation of the constructs employed in this study; Figure S1: Western blot analysis of the proteins expressed from M87-Spastin constructs; Figure S2: Immunocytochemistry assay of full-length Spastin depending on EGF treatment; Figure S3: Immunocytochemistry assays detecting the localization of mutant full-length Spastin proteins; Figure S4: Immunocytochemistry assay of phospho-mimetic M87-Spastin proteins depending on paclitaxel treatment; Spreadsheet S1: Kinase analysis of Spastin’s MBD.
