# Supplementary material for: Spastin Promotes the Migration and Invasion Capability of T98G Glioblastoma Cells by Interacting with Pin1 through Its Microtubule-Binding Domain
Source: Cells. 2023 Jan 27;12(3):427. doi: 10.3390/cells12030427 (PMC9913556; doi:10.3390/cells12030427)
Supplement: Supplementary file 1 [file cells-12-00427-s001.zip › cells-1983945-supplementary.pdf]

Table S1. List of primers used for the creation of the constructs employed in this study.

**Primer Name**	**Sequence**
$Spastin^{M1\&M87}$_F	5′-ACCCAAGCTGGCTAGTTAAGCTTCCACCATGAATTCTCCGGGTGGACGAGG-3′
$Spastin^{M1\&M87}$_R	5′-GCCCTCTAGACTCGAGAACAGTGGTATCTCCAAAGTCCT-3′
Spastin_T292A_F	5′-CCTACCACTCATAAGGGTGCTCCGAAAACAAATAGGA-3′
Spastin_T292A_R	5′-AGCAGGACCAGATCCCTG-3′
Spastin_T303A_F	5′-AGGACAAATAAACCTTCTGCCCCTACAACTGCTACTCGT-3′
Spastin_T303A_R	5′-ATTTGTTTTCGGAGCACCCTT-3′
Spastin_T292D_F	5′-CCTACCACTCATAAGGGTGATCCGAAAACAAATAGGA-3′
Spastin_T292D_R	5′-AGCAGGACCAGATCCCTG-3′
Spastin_T303D_F	5′-AGGACAAATAAACCTTCTGACCCTACAACTGCTACTCGT-3′
Spastin_T303D_R	5′-ATTTGTTTTCGGATCACCCTTATG-3′
$Spastin^{MBD}$_F	5'-ACAAAGCTTGCCACCATGCAACCAGTTTTGCCATTTTCCA-3'
$Spastin^{MBD}$_R	5'-CCCGCTCGAGTGTTCCATTGTCCACAATTTCATTCA-3′
$Spastin^{M87}$_F	5′-CCCAAGCTTATGGCAGCCAAGAGGAGCTC-3′
$Spastin^{M87}$_R	5′-GCCCTCTAGACTCGAGAACAGTGGTATCTCCAAAGTCCT-3′

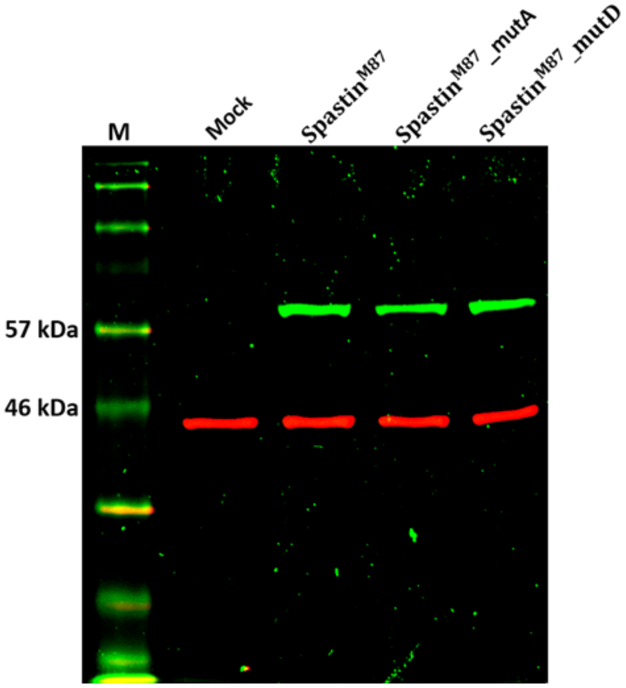

Figure S1. Western blot analysis of the proteins expressed from M87-Spastin constructs. Green bands indicate the expressions of either wild-type M87-Spastin (SpastinM87) or mutant M87-Spastin proteins (SpastinM87_mutA, and SpastinM87_mutD). β-Actin observed as red bands was used as the loading control.

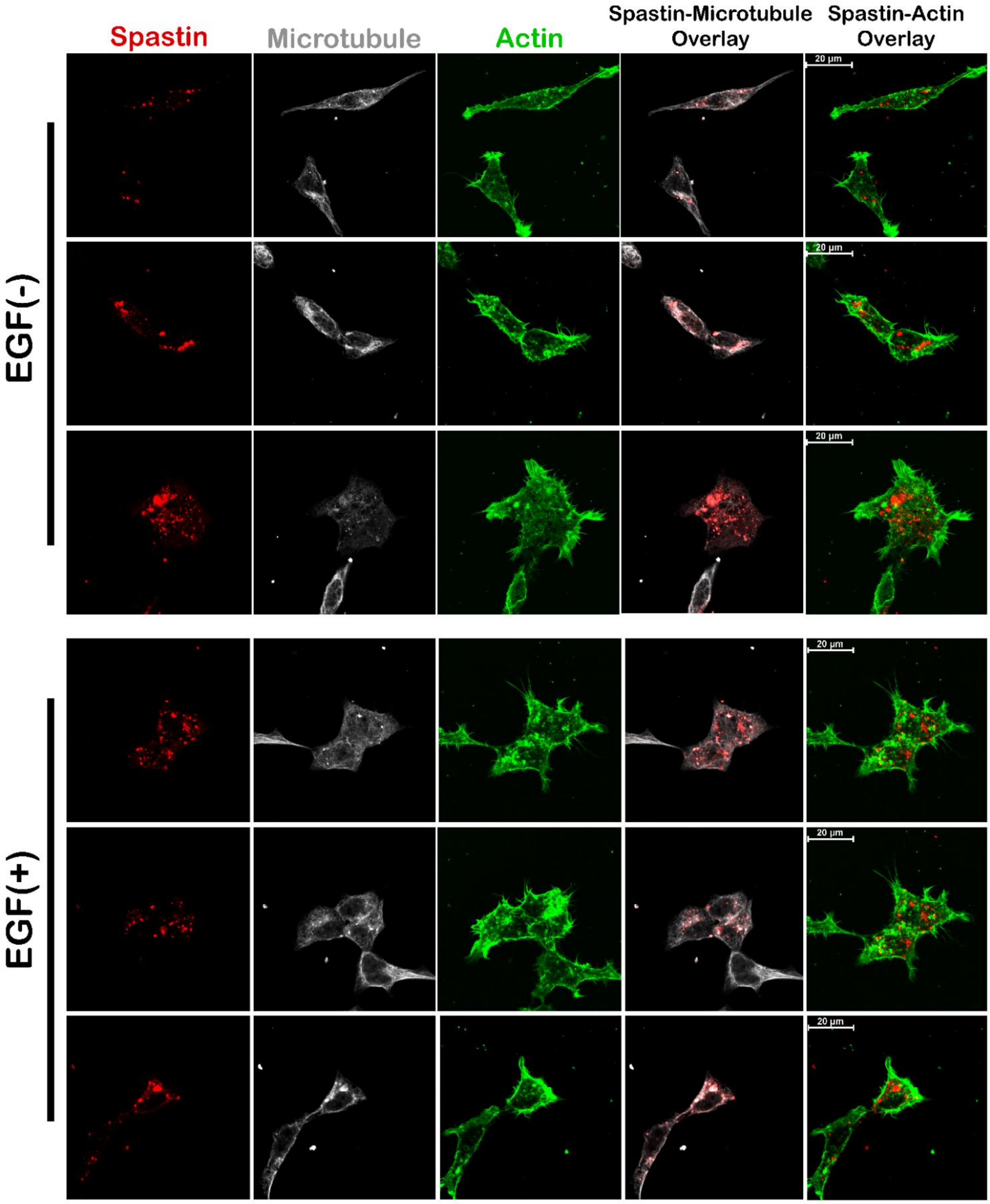

Figure S2. Immunocytochemistry assay of full-length Spastin depending on EGF treatment. T98G cells were transfected with a Spastin$^{M1\&M87}$ vector for 24 h with or without EGF treatment (50 ng/ml). Then, cells were fixed and stained with Myc-tag antibody for Spastin (red), phallacidin for actin filaments (green), and tubulin antibody for microtubule (gray). Cells were visualized by confocal microscopy with a 63x objective.

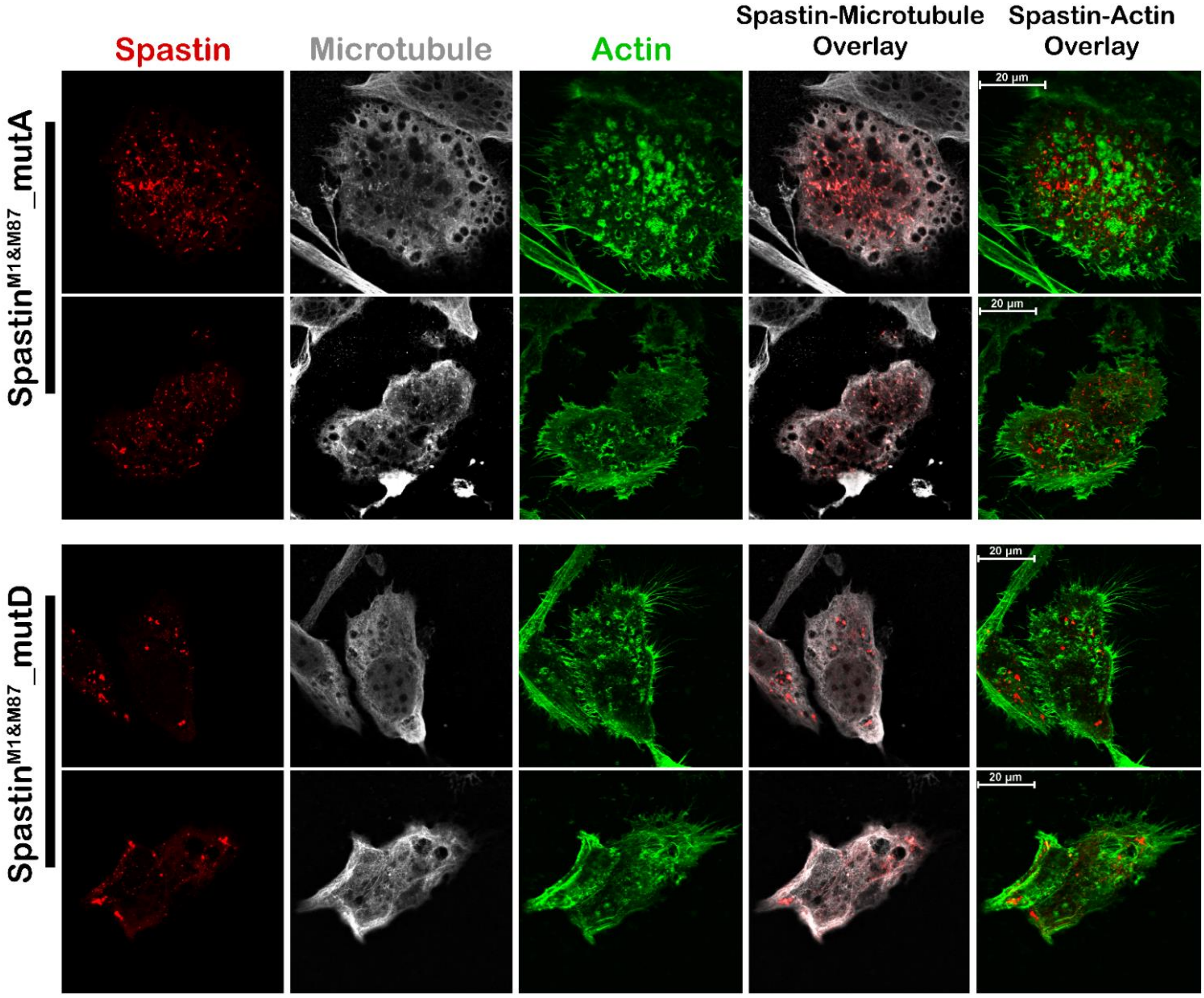

Figure S3. Immunocytochemistry assays detecting the localization of mutant full-length Spastin proteins. T98G cells were transfected with either Spastin$^{M1\&M87}$_mutA or Spastin$^{M1\&M87}$_mutD vectors for 24 h. Then, cells were fixed and stained with Myc-tag antibody for Spastin (red), phallacidin for actin filaments (green), and tubulin antibody for microtubule (gray). Cells were visualized by confocal microscopy with a 63x objective.

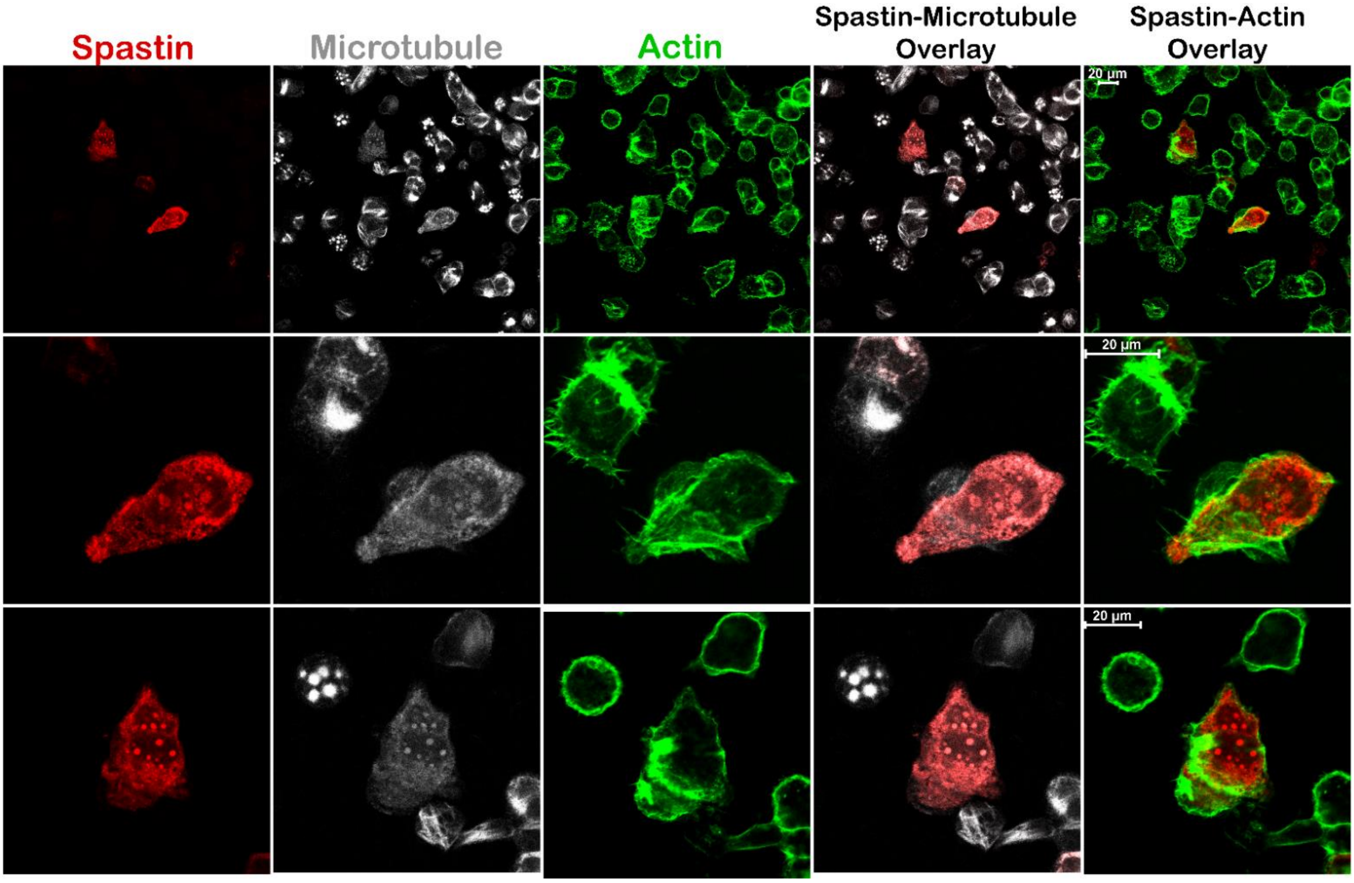

Figure S4. Immunocytochemistry assay of phospho-mimetic M87-Spastin proteins upon paclitaxel treatment. T98G cells were transfected with Spastin^{M87}_mutD vector for 24 h with paclitaxel treatment (10 μM). Then, cells were fixed and stained with Myc-tag antibody for Spastin (red), phallacidin for actin filaments (green), and tubulin antibody for microtubule (gray). Cells were visualized by confocal microscopy with a 40x objective.
